# Crystal structure of 1-hepta­fluoro­tolyl-*closo*-1,2-dicarbadodeca­borane

**DOI:** 10.1107/S2056989019004067

**Published:** 2019-03-29

**Authors:** James D. Watson, Amanda Benton, Hugo Tricas, Georgina M. Rosair, Alan J. Welch

**Affiliations:** a Institute of Chemical Sciences, School of Engineering & Physical Sciences, Heriot-Watt University, Edinburgh, EH14 4AS, UK

**Keywords:** crystal structure, carborane, intra­molecular F⋯H hydrogen-bond

## Abstract

The mol­ecular structure of the title compound 1-(2′,3′,5′,6′-tetra­fluoro-4′-trifluoro­methyl­phen­yl)-*closo*-1,2-dicarbadodeca­borane, C_9_H_11_B_10_F_7_, features an intra­molecular *ortho*-F⋯H2 hydrogen bond [2.11 (2) Å], which is responsible for an orientation of the hepta­fluoro­tolyl substituent in which the plane of the aryl ring nearly eclipses the C1—C2 cage connectivity.

## Abstract 2

The title compound features an intra­molecular hydrogen bond involving the acidic H atom bound to the cage C atom and an *ortho*-F atom of the hepta­fluoro­tolyl substituent.

## Chemical context   

Carborane chemistry continues to be an area of intense academic inter­est but also one that has both potential and real applications in a wide variety of fields, with a particular blossoming of such applications over the last two decades (Grimes, 2016 ▸). Two important factors driving studies into the synthesis and properties of novel carborane compounds for a vast array of applications are the high chemical and thermal stabilities of such species and the relative ease of their deriv­atization. Several years ago we described a family of doubly substituted *closo*-C_2_B_10_ carboranes bearing fluorinated aryl groups (Tricas *et al.*, 2011 ▸). Our comprehensive (synthetic, spectroscopic, structural, electrochemical and computational) study focused primarily on the stabilization of the reduced form of the carboranes by the presence of the strongly electron-withdrawing fluoroaryl groups, and the study has attracted considerable attention from those working in the related field of carborane photophysics (*e.g.* Van Nghia *et al.*, 2018 ▸; Marsh *et al.*, 2018 ▸). Very recently we have reported the first examples of substituted carboranes as components of inter­molecular frustrated Lewis pairs (FLPs; Benton *et al.*, 2018 ▸). In this field the ability to fine-tune the Lewis acidity or basicity of a functional group on a carborane support by the electron-withdrawing or electron-donating characteristics of a second substituent on the carborane is of potential importance in using these FLPs as catalysts. Herein we report the synthesis and crystal structure of [1-(4′-F_3_CC_6_F_4_)-*closo*-1,2-C_2_B_10_H_11_], a singly substituted fluoroaryl carborane with the potential for further derivatization.

## Structural commentary   

**Figure 1:**
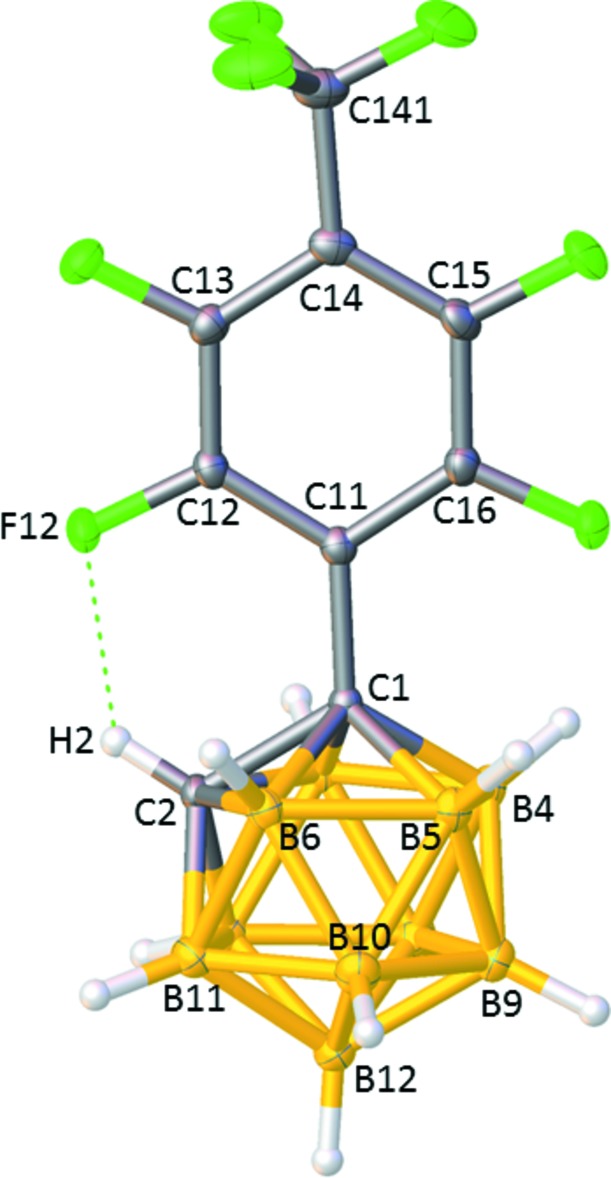
The mol­ecular structure of [1-(4′-F_3_CC_6_F_4_)-*closo*-1,2-C_2_B_10_H_11_] with key atoms labelled. Displacement ellipsoids are drawn at the 50% probability level, except for H atoms. The hydrogen bond between F12 and H2 is shown as a dotted line.

H atoms bound to C in *closo* carboranes are protonic in nature (Grimes, 2016 ▸) and the strongly electron-withdrawing nature of the perfluoro­tolyl substituent on C1 renders the H atom on C2 in [1-(4′-F_3_CC_6_F_4_)-*closo*-1,2-C_2_B_10_H_11_] particularly protonic, as evidenced by its high-frequency ^1^H NMR chemical shift (δ 4.88 ppm). This makes the C1H1 unit a strong hydrogen-bond donor and results in the most striking feature of the structure (Fig. 1 ▸), the intra­molecular hydrogen bond between F12 and H2. Mol­ecular dimensions for the hydrogen bond are given in Table 1 ▸ and are complemented by the near-tetra­hedral angle C12—F12⋯H2 = 108.4 (6) °. This hydrogen bond is responsible for the orientation of the 4′-F_3_CC_6_F_4_ substituent with respect to the carborane in the solid state, defined by the torsion angle C2—C1—C11—C12 = 9.6 (2)°, in which the plane of the aryl ring almost eclipses the C1—C2 connectivity.
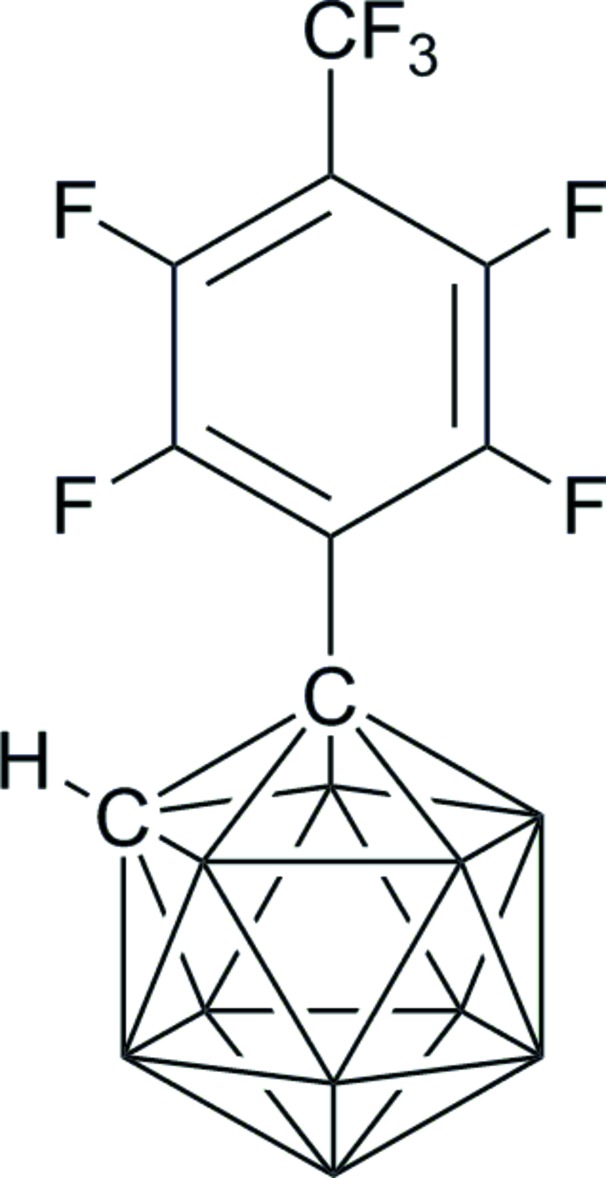


The only other [1-(*ortho*-F-ar­yl)-*closo*-1,2-C_2_B_10_H_11_] species to have been studied crystallographically is that with a 2′-fluoro-4′-(9′′-phenanthren­yl) substituent (Tu *et al.*, 2017 ▸). In this species there is an inter­molecular F⋯C_cage_H hydrogen-bond, 2.091 (4) Å, between the two crystallographically independent mol­ecules in the asymmetric fraction of the unit cell, although the situation is somewhat complicated by partial disorder of both F atoms. The C1—C2 distance in [1-(4′-F_3_CC_6_F_4_)-*closo*-1,2-C_2_B_10_H_11_], 1.660 (2) Å, stands good comparison with that in [1-Ph-*closo*-1,2-C_2_B_10_H_11_] [α polymorph, 1.640 (5) Å, Brain *et al.*, 1996 ▸; β polymorph, 1.649 (2) Å, Thomas *et al.*, 1996 ▸]. Dimensions within the 4′-F_3_CC_6_F_4_ substituent are fully consistent with those in [1-(4′-F_3_CC_6_F_4_)-2-Ph-*closo*-1,2-C_2_B_10_H_10_], [1,2-(4′-F_3_CC_6_F_4_)_2_-*closo*-1,2-C_2_B_10_H_10_], [1,7-(4′-F_3_CC_6_F_4_)_2_-*closo*-1,7-C_2_B_10_H_10_] and [1,12-(4′-F_3_CC_6_F_4_)_2_-*closo*-1,12-C_2_B_10_H_10_] (Tricas *et al.*, 2011 ▸).

## Supra­molecular features   

**Figure 2:**
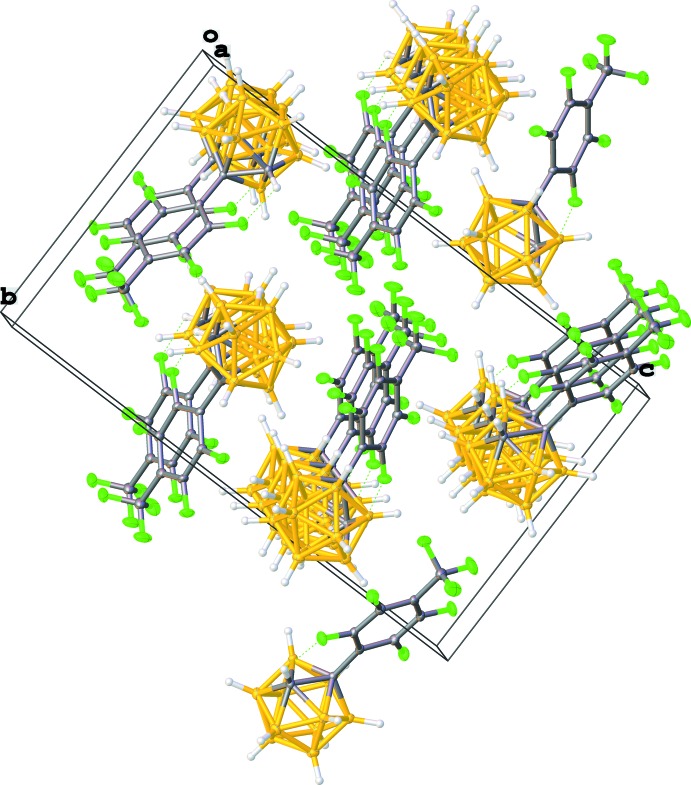
Unit cell of [1-(4′-F_3_CC_6_F_4_)-*closo*-1,2-C_2_B_10_H_11_] in a view along [100].

Mol­ecules pack in ribbons parallel to the crystallographic *a* axis, but there are no significant inter­molecular contacts either within or between these ribbons. A view of the crystal packing along [100] is shown in Fig. 2 ▸.

## Database survey   

A search of the Cambridge Structural Database (CSD, 2019 release; Groom *et al.*, 2016 ▸) yielded 384 examples of [*C*-aryl-*closo*-1,2-C_2_B_10_] carboranes. However, this number drops to 63 if the second cage C atom is not substituted, *i.e.* structures of the type [1-aryl-*closo*-1,2-C_2_B_10_H_11_]. Furthermore, there are only two reported structural studies of cases where the aryl ring is at least partially fluorinated, the aforementioned 2′-fluoro-4′-(9′′-phenanthren­yl) species (Tu *et al.*, 2017 ▸) and [1-(4′-C_6_H_4_F)-*closo*-1,2-C_2_B_10_H_11_] (Clegg, 2016 ▸). Removing the condition that the second cage C atom is not substituted affords 19 further examples of fluoroaryl derivatives of [*closo*-1,2-C_2_B_10_H_11_]. There are only three examples where a 4′-F_3_CC_6_F_4_ substituent is attached to a [*closo*-1,2-C_2_B_10_] cage, two of which result from our laboratories (Tricas *et al.*, 2011 ▸) and the other from Lee *et al.* (2017 ▸).

## Synthesis and crystallization   

Under dry N_2_ and using anhydrous, degassed solvents, [*closo*-1,2-C_2_B_10_H_12_] (0.75 g, 5.2 mmol) was dissolved in a 1:1 mixture of toluene and diethyl ether (40 mL). The colourless solution was cooled to 273 K before *n*-BuLi (3.58 mL of a 1.6 *M* solution in hexa­nes, 5.73 mmol, 1.1 equiv.) was added dropwise over the course of 2 min. whilst stirring vigorously. The solution was warmed to room temperature and changed from colourless to yellow. After further stirring for 1 h the solution was cooled to 273 K, resulting in a white suspension. Whilst stirring vigorously, octa­fluoro­toluene (0.74 mL, 5.2 mmol, 1.0 equiv.) was added dropwise over the course of 1 min., causing the solution to turn from yellow to deep red. The solution was stirred for 4 h at room temperature and then quenched with saturated [NH_4_]Cl (aq., 20 mL). The organic layer was isolated and the aqueous phase extracted with Et_2_O (3 × 20 mL). The organic phases were combined and reduced in volume *in vacuo* to yield a brown residue. Products were isolated by column chromatography on silica eluting with 313–333 K petroleum ether to give both the target compound [1-(4′-F_3_CC_6_F_4_)-*closo*-1,2-C_2_B_10_H_11_] (*R*_f_ = 0.27, 0.57 g, 30% yield) and the disubstituted species [1,2-(4′-F_3_CC_6_F_4_)_2_-*closo*-1,2-C_2_B_10_H_10_] (*R*_f_ = 0.37, 0.33 g, 11% yield, Tricas *et al.*, 2011 ▸) as colourless solids once evacuated to dryness.

C_9_H_11_B_10_F_7_ requires; C 30.0, H 3.08. Found; C 30.5, H 2.83%. ^1^H NMR (CDCl_3_, 400.1 MHz, 298 K, δ): 4.88 (*br. s*, 1H, C*H*_cage_). ^11^B{^1^H} NMR (CDCl_3_, 128.4 MHz, 298 K, δ): −0.32 (1B), −1.80 (1B), −8.06 (2B), −9.62 (2B), −11.17 (2B), −12.89 (2B). ^19^F NMR (CDCl_3_, 376.5 MHz, 298 K, δ): −56.72 (*t*, 3F, *J*_FF_ = 21.3 Hz, C*F*_3_), −135.17 (*br. s*, 2F, *F*_ortho_), −137.26 (*m*, 2F, *F*_meta_). Crystals of [1-(4′-F_3_CC_6_F_4_)-*closo*-1,2-C_2_B_10_H_11_] suitable for a single-crystal *X*-ray diffraction study were grown from the slow evaporation of a 313–333 K petroleum ether solution of the product.

## Refinement   

Crystal data, data collection and structure refinement details are summarized in Table 2 ▸. The cage C atom (C2) not carrying the substituent was distinguished from B atoms by both the *Vertex–Centroid Distance* (McAnaw *et al.*, 2013 ▸) and *Boron–Hydrogen Distance* (McAnaw *et al.*, 2014 ▸) methods. Cage H atoms were located from difference-Fourier maps and allowed positional refinement, with *U*_iso_(H) = 1.2*U*_eq_(B or C). Five poorly fitting reflections were omitted which marginally decreased the *R*-factor and standard uncertainties from the previous refinement.

## Supplementary Material

Crystal structure: contains datablock(s) I. DOI: 10.1107/S2056989019004067/lh5894sup1.cif

Structure factors: contains datablock(s) I. DOI: 10.1107/S2056989019004067/lh5894Isup2.hkl

Supporting information file. DOI: 10.1107/S2056989019004067/lh5894Isup3.mol

CCDC reference: 1905663

Additional supporting information:  crystallographic information; 3D view; checkCIF report

## Figures and Tables

**Table 1:** Hydrogen-bond geometry (Å, °)

*D*—H⋯*A*	*D*—H	H⋯*A*	*D*⋯*A*	*D*—H⋯*A*
C2—H2⋯F12	0.91 (2)	2.11 (2)	2.7436 (19)	126 (2)

**Table 2:** Experimental details

Crystal data	
Chemical formula	C_9_H_11_B_10_F_7_
*M*_r_	360.28
Crystal system, space group	Orthorhombic, *P*2_1_2_1_2_1_
Temperature (K)	120
*a*, *b*, *c* (Å)	6.7872 (2), 11.6926 (3), 19.4863 (5)
*V* (Å^3^)	1546.43 (7)
*Z*	4
Radiation type	Mo *K*α
μ (mm^−1^)	0.14
Crystal size (mm)	0.30 × 0.21 × 0.10

Data collection	
Diffractometer	Rigaku Oxford Diffreaction SuperNova
Absorption correction	Multi-scan (*CrysAlis PRO*; Rigaku OD, 2018 ▸)
*T*_min_, *T*_max_	0.907, 1.000
No. of measured, independent and observed [*I* > 2σ(*I*)] reflections	40258, 5615, 5190
*R*_int_	0.041
(sin θ/λ)_max_ (Å^−1^)	0.768

Refinement	
*R*[*F* ^2^ > 2σ(*F* ^2^)], *wR*(*F* ^2^), *S*	0.039, 0.092, 1.15
No. of reflections	5615
No. of parameters	268
H-atom treatment	Only H-atom coordinates refined
Δρ_max_, Δρ_min_ (e Å^−3^)	0.32, −0.24
Absolute structure	Flack *x* determined using 1991 quotients [(*I* ^+^)−(*I* ^−^)]/[(*I* ^+^)+(*I* ^−^)] (Parsons et al., 2013 ▸)
Absolute structure parameter	−0.03 (14)

Computer programs: *CrysAlis PRO* (Rigaku OD, 2018 ▸), *SHELXT* (Sheldrick, 2015*a* ▸), *SHELXL* (Sheldrick, 2015*b* ▸) and *OLEX2* (Dolomanov *et al.*, 2009 ▸).

## supplementary crystallographic information

### Crystal data

**Table:**

C_9_H_11_B_10_F_7_	*D*_x_ = 1.547 Mg m^−^^3^
*M**_r_* = 360.28	Mo *K*α radiation, λ = 0.71073 Å
Orthorhombic, *P*2_1_2_1_2_1_	Cell parameters from 14204 reflections
*a* = 6.7872 (2) Å	θ = 3.6–32.3°
*b* = 11.6926 (3) Å	µ = 0.14 mm^−^^1^
*c* = 19.4863 (5) Å	*T* = 120 K
*V* = 1546.43 (7) Å^3^	Block, colourless
*Z* = 4	0.30 × 0.21 × 0.10 mm
*F*(000) = 712	

### Data collection

**Table:**

Rigaku Oxford Diffreaction SuperNova diffractometer	5615 independent reflections
Radiation source: micro-focus sealed X-ray tube, SuperNova (Mo) X-ray Source	5190 reflections with *I* > 2σ(*I*)
Mirror monochromator	*R*_int_ = 0.041
Detector resolution: 5.1574 pixels mm^-1^	θ_max_ = 33.1°, θ_min_ = 3.2°
ω scans	*h* = −10→10
Absorption correction: multi-scan (CrysAlis PRO; Rigaku OD, 2018)	*k* = −17→17
*T*_min_ = 0.907, *T*_max_ = 1.000	*l* = −28→29
40258 measured reflections	

### Refinement

**Table:**

Refinement on *F*^2^	Hydrogen site location: difference Fourier map
Least-squares matrix: full	Only H-atom coordinates refined
*R*[*F*^2^ > 2σ(*F*^2^)] = 0.039	*w* = 1/[σ^2^(*F*_o_^2^) + (0.0409*P*)^2^ + 0.2387*P*] where *P* = (*F*_o_^2^ + 2*F*_c_^2^)/3
*wR*(*F*^2^) = 0.092	(Δ/σ)_max_ < 0.001
*S* = 1.15	Δρ_max_ = 0.32 e Å^−^^3^
5615 reflections	Δρ_min_ = −0.24 e Å^−^^3^
268 parameters	Absolute structure: Flack *x* determined using 1991 quotients [(*I*^+^)-(*I*^-^)]/[(*I*^+^)+(*I*^-^)] (Parsons et al., 2013)
0 restraints	Absolute structure parameter: −0.03 (14)
Primary atom site location: dual	

### Special details

**Table:**

Geometry. All esds (except the esd in the dihedral angle between two l.s. planes) are estimated using the full covariance matrix. The cell esds are taken into account individually in the estimation of esds in distances, angles and torsion angles; correlations between esds in cell parameters are only used when they are defined by crystal symmetry. An approximate (isotropic) treatment of cell esds is used for estimating esds involving l.s. planes.

### Fractional atomic coordinates and isotropic or equivalent isotropic displacement parameters (Å^2^)

**Table:**

	*x*	*y*	*z*	*U*_iso_*/*U*_eq_	
C1	0.4255 (2)	0.75630 (14)	0.63450 (7)	0.0127 (3)	
C2	0.3379 (2)	0.84949 (14)	0.69108 (8)	0.0144 (3)	
H2	0.239 (3)	0.825 (2)	0.7189 (11)	0.017*	
B3	0.5657 (3)	0.79020 (16)	0.70696 (9)	0.0150 (3)	
H3	0.569 (4)	0.731 (2)	0.7447 (11)	0.018*	
B4	0.6746 (3)	0.78014 (17)	0.62414 (9)	0.0152 (3)	
H4	0.760 (3)	0.7102 (19)	0.6185 (11)	0.018*	
B5	0.4984 (3)	0.82791 (16)	0.56196 (9)	0.0157 (3)	
H5	0.481 (3)	0.7834 (19)	0.5125 (11)	0.019*	
B6	0.2792 (3)	0.86920 (16)	0.60596 (9)	0.0156 (3)	
H6	0.137 (3)	0.848 (2)	0.5881 (11)	0.019*	
B7	0.5140 (3)	0.93737 (17)	0.72239 (9)	0.0173 (3)	
H7	0.498 (3)	0.960 (2)	0.7758 (11)	0.021*	
B8	0.7317 (3)	0.89674 (17)	0.67875 (10)	0.0172 (3)	
H8	0.868 (4)	0.904 (2)	0.7027 (11)	0.021*	
B9	0.6915 (3)	0.92010 (17)	0.58946 (9)	0.0174 (3)	
H9	0.810 (3)	0.940 (2)	0.5576 (11)	0.021*	
B10	0.4473 (3)	0.97497 (17)	0.57793 (10)	0.0191 (3)	
H10	0.411 (4)	1.030 (2)	0.5388 (11)	0.023*	
C11	0.3333 (2)	0.63954 (14)	0.62898 (7)	0.0133 (3)	
B11	0.3386 (3)	0.98580 (16)	0.66084 (10)	0.0178 (3)	
H11	0.223 (4)	1.039 (2)	0.6735 (11)	0.021*	
F12	0.06277 (15)	0.67898 (9)	0.70430 (5)	0.0194 (2)	
C12	0.1573 (2)	0.60768 (14)	0.66176 (8)	0.0142 (3)	
B12	0.5930 (3)	1.01793 (17)	0.65039 (10)	0.0185 (3)	
H12	0.642 (4)	1.101 (2)	0.6567 (12)	0.022*	
F13	−0.09989 (16)	0.48161 (9)	0.68394 (5)	0.0216 (2)	
C13	0.0708 (3)	0.50228 (14)	0.65191 (8)	0.0160 (3)	
C14	0.1543 (3)	0.41874 (14)	0.61068 (8)	0.0171 (3)	
F15	0.4241 (2)	0.37422 (9)	0.53809 (6)	0.0269 (3)	
C15	0.3300 (3)	0.44732 (14)	0.57878 (8)	0.0177 (3)	
F16	0.58167 (16)	0.57283 (9)	0.55240 (5)	0.0215 (2)	
C16	0.4149 (3)	0.55452 (14)	0.58724 (8)	0.0157 (3)	
F17	−0.1264 (2)	0.31645 (11)	0.57675 (7)	0.0366 (3)	
F18	0.0274 (2)	0.25723 (10)	0.66597 (6)	0.0341 (3)	
F19	0.1512 (2)	0.23058 (11)	0.56619 (7)	0.0382 (3)	
C141	0.0518 (3)	0.30462 (15)	0.60420 (9)	0.0227 (4)	

### Atomic displacement parameters (Å^2^)

**Table:**

	*U*^11^	*U*^22^	*U*^33^	*U*^12^	*U*^13^	*U*^23^
C1	0.0121 (6)	0.0145 (7)	0.0115 (6)	0.0011 (5)	0.0001 (5)	0.0002 (5)
C2	0.0142 (6)	0.0147 (7)	0.0143 (6)	−0.0005 (6)	0.0025 (6)	−0.0028 (5)
B3	0.0150 (7)	0.0177 (8)	0.0125 (7)	0.0003 (6)	−0.0021 (6)	−0.0009 (6)
B4	0.0119 (7)	0.0192 (8)	0.0145 (7)	0.0015 (7)	0.0001 (6)	−0.0002 (6)
B5	0.0157 (7)	0.0186 (8)	0.0128 (7)	0.0018 (7)	−0.0001 (6)	0.0017 (6)
B6	0.0141 (8)	0.0162 (8)	0.0164 (8)	0.0034 (6)	−0.0007 (6)	0.0001 (6)
B7	0.0176 (8)	0.0184 (8)	0.0158 (7)	−0.0029 (7)	0.0014 (6)	−0.0030 (6)
B8	0.0139 (8)	0.0199 (8)	0.0179 (8)	−0.0027 (7)	0.0001 (6)	−0.0021 (7)
B9	0.0153 (8)	0.0201 (8)	0.0167 (8)	−0.0007 (7)	0.0025 (6)	0.0010 (6)
B10	0.0204 (8)	0.0186 (8)	0.0183 (8)	0.0024 (7)	0.0002 (7)	0.0040 (6)
C11	0.0138 (6)	0.0152 (7)	0.0111 (6)	0.0004 (6)	−0.0010 (5)	−0.0009 (5)
B11	0.0175 (8)	0.0150 (7)	0.0208 (8)	0.0016 (7)	0.0012 (7)	−0.0014 (7)
F12	0.0153 (4)	0.0195 (5)	0.0234 (5)	−0.0007 (4)	0.0064 (4)	−0.0067 (4)
C12	0.0150 (7)	0.0159 (7)	0.0116 (6)	0.0012 (6)	0.0001 (5)	−0.0020 (5)
B12	0.0171 (8)	0.0171 (8)	0.0213 (8)	−0.0013 (7)	0.0020 (7)	−0.0011 (7)
F13	0.0180 (5)	0.0231 (5)	0.0239 (5)	−0.0056 (4)	0.0047 (4)	−0.0001 (4)
C13	0.0164 (7)	0.0178 (7)	0.0137 (6)	−0.0014 (6)	0.0002 (6)	0.0007 (5)
C14	0.0230 (8)	0.0152 (7)	0.0131 (6)	−0.0013 (6)	−0.0033 (6)	−0.0001 (5)
F15	0.0349 (6)	0.0197 (5)	0.0262 (5)	0.0019 (5)	0.0096 (5)	−0.0102 (4)
C15	0.0244 (8)	0.0156 (7)	0.0130 (6)	0.0021 (6)	0.0018 (6)	−0.0028 (5)
F16	0.0204 (5)	0.0231 (5)	0.0211 (5)	−0.0007 (4)	0.0087 (4)	−0.0055 (4)
C16	0.0167 (7)	0.0169 (7)	0.0137 (6)	0.0007 (6)	0.0025 (6)	−0.0005 (5)
F17	0.0359 (7)	0.0292 (6)	0.0447 (7)	−0.0129 (5)	−0.0165 (6)	0.0038 (6)
F18	0.0577 (9)	0.0216 (5)	0.0229 (5)	−0.0110 (6)	−0.0015 (6)	0.0062 (4)
F19	0.0506 (8)	0.0214 (6)	0.0425 (7)	−0.0066 (6)	0.0098 (7)	−0.0142 (5)
C141	0.0311 (10)	0.0181 (8)	0.0188 (7)	−0.0056 (7)	−0.0030 (7)	0.0000 (6)

### Geometric parameters (Å, º)

**Table:**

C1—C2	1.660 (2)	B7—B11	1.782 (3)
C1—B3	1.748 (2)	B7—B12	1.773 (3)
C1—B4	1.726 (2)	B8—H8	1.04 (2)
C1—B5	1.716 (2)	B8—B9	1.782 (3)
C1—B6	1.743 (2)	B8—B12	1.789 (3)
C1—C11	1.506 (2)	B9—H9	1.04 (2)
C2—H2	0.91 (2)	B9—B10	1.792 (3)
C2—B3	1.723 (3)	B9—B12	1.779 (3)
C2—B6	1.721 (2)	B10—H10	1.03 (2)
C2—B7	1.690 (2)	B10—B11	1.781 (3)
C2—B11	1.699 (3)	B10—B12	1.796 (3)
B3—H3	1.01 (2)	C11—C12	1.405 (2)
B3—B4	1.779 (2)	C11—C16	1.399 (2)
B3—B7	1.782 (3)	B11—H11	1.03 (2)
B3—B8	1.767 (3)	B11—B12	1.779 (3)
B4—H4	1.01 (2)	F12—C12	1.3394 (18)
B4—B5	1.792 (3)	C12—C13	1.378 (2)
B4—B8	1.772 (3)	B12—H12	1.03 (2)
B4—B9	1.774 (3)	F13—C13	1.338 (2)
B5—H5	1.10 (2)	C13—C14	1.386 (2)
B5—B6	1.784 (3)	C14—C15	1.386 (3)
B5—B9	1.780 (3)	C14—C141	1.510 (2)
B5—B10	1.782 (3)	F15—C15	1.3294 (19)
B6—H6	1.06 (2)	C15—C16	1.389 (2)
B6—B10	1.769 (3)	F16—C16	1.3372 (19)
B6—B11	1.779 (3)	F17—C141	1.330 (2)
B7—H7	1.08 (2)	F18—C141	1.335 (2)
B7—B8	1.770 (3)	F19—C141	1.324 (2)
			
C2—C1—B3	60.65 (10)	B12—B7—B3	108.66 (13)
C2—C1—B4	108.82 (13)	B12—B7—H7	131.8 (13)
C2—C1—B5	109.28 (12)	B12—B7—B11	60.04 (11)
C2—C1—B6	60.71 (10)	B3—B8—B4	60.34 (10)
B4—C1—B3	61.60 (10)	B3—B8—B7	60.50 (11)
B4—C1—B6	113.49 (12)	B3—B8—H8	118.9 (13)
B5—C1—B3	113.44 (12)	B3—B8—B9	108.31 (13)
B5—C1—B4	62.76 (10)	B3—B8—B12	108.60 (14)
B5—C1—B6	62.08 (10)	B4—B8—H8	121.7 (13)
B6—C1—B3	113.34 (12)	B4—B8—B9	59.89 (10)
C11—C1—C2	119.58 (13)	B4—B8—B12	107.99 (13)
C11—C1—B3	119.30 (12)	B7—B8—B4	108.21 (13)
C11—C1—B4	123.05 (14)	B7—B8—H8	120.4 (13)
C11—C1—B5	120.23 (12)	B7—B8—B9	107.47 (13)
C11—C1—B6	115.29 (13)	B7—B8—B12	59.76 (11)
C1—C2—H2	117.1 (15)	B9—B8—H8	124.2 (13)
C1—C2—B3	62.21 (10)	B9—B8—B12	59.77 (11)
C1—C2—B6	62.01 (9)	B12—B8—H8	123.0 (13)
C1—C2—B7	112.67 (13)	B4—B9—B5	60.56 (10)
C1—C2—B11	112.60 (12)	B4—B9—B8	59.78 (11)
B3—C2—H2	115.4 (15)	B4—B9—H9	118.6 (13)
B6—C2—H2	116.6 (14)	B4—B9—B10	108.56 (13)
B6—C2—B3	115.76 (12)	B4—B9—B12	108.33 (13)
B7—C2—H2	119.7 (14)	B5—B9—B8	108.29 (13)
B7—C2—B3	62.93 (11)	B5—B9—H9	121.5 (13)
B7—C2—B6	115.47 (13)	B5—B9—B10	59.85 (11)
B7—C2—B11	63.45 (11)	B8—B9—H9	119.8 (12)
B11—C2—H2	120.2 (15)	B8—B9—B10	108.59 (13)
B11—C2—B3	115.93 (13)	B10—B9—H9	124.2 (13)
B11—C2—B6	62.67 (10)	B12—B9—B5	108.27 (13)
C1—B3—H3	116.6 (13)	B12—B9—B8	60.30 (11)
C1—B3—B4	58.57 (9)	B12—B9—H9	123.2 (13)
C1—B3—B7	104.36 (12)	B12—B9—B10	60.38 (11)
C1—B3—B8	104.81 (12)	B5—B10—B9	59.74 (11)
C2—B3—C1	57.14 (9)	B5—B10—H10	121.3 (13)
C2—B3—H3	115.2 (14)	B5—B10—B12	107.46 (13)
C2—B3—B4	103.69 (12)	B6—B10—B5	60.31 (10)
C2—B3—B7	57.65 (10)	B6—B10—B9	107.91 (13)
C2—B3—B8	103.45 (13)	B6—B10—H10	120.6 (14)
B4—B3—H3	127.5 (13)	B6—B10—B11	60.16 (11)
B4—B3—B7	107.38 (13)	B6—B10—B12	107.93 (13)
B7—B3—H3	122.7 (13)	B9—B10—H10	122.7 (14)
B8—B3—H3	134.0 (14)	B9—B10—B12	59.47 (11)
B8—B3—B4	59.97 (11)	B11—B10—B5	107.93 (13)
B8—B3—B7	59.82 (11)	B11—B10—B9	107.16 (13)
C1—B4—B3	59.83 (10)	B11—B10—H10	122.0 (13)
C1—B4—H4	116.4 (13)	B11—B10—B12	59.65 (11)
C1—B4—B5	58.35 (10)	B12—B10—H10	122.9 (14)
C1—B4—B8	105.56 (13)	C12—C11—C1	124.15 (14)
C1—B4—B9	104.88 (12)	C16—C11—C1	121.42 (14)
B3—B4—H4	113.0 (12)	C16—C11—C12	114.37 (15)
B3—B4—B5	108.39 (13)	C2—B11—B6	59.27 (10)
B5—B4—H4	124.2 (12)	C2—B11—B7	58.03 (10)
B8—B4—B3	59.69 (10)	C2—B11—B10	104.42 (13)
B8—B4—H4	124.3 (13)	C2—B11—H11	119.0 (13)
B8—B4—B5	108.19 (14)	C2—B11—B12	103.91 (13)
B8—B4—B9	60.34 (11)	B6—B11—B7	108.20 (13)
B9—B4—B3	108.15 (13)	B6—B11—B10	59.59 (11)
B9—B4—H4	132.0 (13)	B6—B11—H11	116.0 (13)
B9—B4—B5	59.87 (11)	B6—B11—B12	108.23 (13)
C1—B5—B4	58.89 (10)	B7—B11—H11	122.5 (13)
C1—B5—H5	117.4 (12)	B10—B11—B7	108.13 (13)
C1—B5—B6	59.70 (10)	B10—B11—H11	125.1 (13)
C1—B5—B9	105.07 (12)	B12—B11—B7	59.71 (11)
C1—B5—B10	105.72 (12)	B12—B11—B10	60.59 (11)
B4—B5—H5	121.0 (12)	B12—B11—H11	129.4 (13)
B6—B5—B4	108.42 (12)	F12—C12—C11	121.58 (14)
B6—B5—H5	117.4 (12)	F12—C12—C13	116.01 (14)
B9—B5—B4	59.57 (11)	C13—C12—C11	122.40 (14)
B9—B5—H5	128.9 (12)	B7—B12—B8	59.59 (11)
B9—B5—B6	107.79 (13)	B7—B12—B9	107.47 (14)
B9—B5—B10	60.41 (11)	B7—B12—B10	107.89 (14)
B10—B5—B4	108.22 (13)	B7—B12—B11	60.24 (11)
B10—B5—H5	126.0 (12)	B7—B12—H12	120.2 (13)
B10—B5—B6	59.49 (11)	B8—B12—B10	108.13 (13)
C1—B6—B5	58.22 (9)	B8—B12—H12	122.4 (14)
C1—B6—H6	116.7 (13)	B9—B12—B8	59.94 (11)
C1—B6—B10	105.12 (13)	B9—B12—B10	60.16 (11)
C1—B6—B11	105.05 (12)	B9—B12—H12	124.1 (14)
C2—B6—C1	57.27 (9)	B10—B12—H12	122.3 (13)
C2—B6—B5	103.54 (12)	B11—B12—B8	107.95 (13)
C2—B6—H6	119.8 (12)	B11—B12—B9	107.79 (13)
C2—B6—B10	104.01 (13)	B11—B12—B10	59.76 (11)
C2—B6—B11	58.06 (10)	B11—B12—H12	119.9 (14)
B5—B6—H6	122.8 (12)	C12—C13—C14	122.48 (15)
B10—B6—B5	60.20 (11)	F13—C13—C12	117.73 (14)
B10—B6—H6	130.7 (12)	F13—C13—C14	119.80 (15)
B10—B6—B11	60.25 (11)	C13—C14—C15	116.21 (15)
B11—B6—B5	107.92 (13)	C13—C14—C141	118.91 (16)
B11—B6—H6	125.7 (13)	C15—C14—C141	124.88 (16)
C2—B7—B3	59.42 (10)	C14—C15—C16	121.42 (15)
C2—B7—H7	115.0 (13)	F15—C15—C14	121.74 (16)
C2—B7—B8	104.70 (13)	F15—C15—C16	116.84 (15)
C2—B7—B11	58.52 (11)	C15—C16—C11	123.09 (15)
C2—B7—B12	104.55 (13)	F16—C16—C11	121.11 (15)
B3—B7—H7	114.5 (13)	F16—C16—C15	115.80 (14)
B3—B7—B11	108.96 (13)	F17—C141—C14	111.14 (15)
B8—B7—B3	59.68 (11)	F17—C141—F18	107.02 (17)
B8—B7—H7	127.8 (13)	F18—C141—C14	110.39 (14)
B8—B7—B11	108.64 (13)	F19—C141—C14	112.96 (16)
B8—B7—B12	60.65 (11)	F19—C141—F17	107.84 (15)
B11—B7—H7	120.4 (13)	F19—C141—F18	107.23 (16)
			
C1—C2—B3—B4	37.23 (11)	B5—B6—B11—C2	−95.07 (13)
C1—C2—B3—B7	139.34 (13)	B5—B6—B11—B7	−62.62 (16)
C1—C2—B3—B8	99.11 (12)	B5—B6—B11—B10	38.15 (12)
C1—C2—B6—B5	−36.99 (11)	B5—B6—B11—B12	0.60 (17)
C1—C2—B6—B10	−99.16 (13)	B5—B9—B10—B6	37.70 (12)
C1—C2—B6—B11	−139.86 (13)	B5—B9—B10—B11	101.12 (14)
C1—C2—B7—B3	−38.66 (12)	B5—B9—B10—B12	138.37 (13)
C1—C2—B7—B8	1.77 (17)	B5—B9—B12—B7	63.77 (16)
C1—C2—B7—B11	104.88 (14)	B5—B9—B12—B8	101.08 (14)
C1—C2—B7—B12	64.68 (16)	B5—B9—B12—B10	−37.22 (12)
C1—C2—B11—B6	38.07 (12)	B5—B9—B12—B11	0.22 (17)
C1—C2—B11—B7	−104.99 (14)	B5—B10—B11—C2	2.09 (18)
C1—C2—B11—B10	−2.38 (18)	B5—B10—B11—B6	−38.21 (13)
C1—C2—B11—B12	−65.08 (16)	B5—B10—B11—B7	62.68 (17)
C1—B3—B4—B5	33.61 (12)	B5—B10—B11—B12	100.14 (15)
C1—B3—B4—B8	134.37 (13)	B5—B10—B12—B7	−63.30 (16)
C1—B3—B4—B9	97.01 (13)	B5—B10—B12—B8	−0.31 (17)
C1—B3—B7—C2	34.40 (11)	B5—B10—B12—B9	36.98 (12)
C1—B3—B7—B8	−98.99 (13)	B5—B10—B12—B11	−100.94 (14)
C1—B3—B7—B11	2.00 (16)	B6—C1—C2—B3	−146.76 (13)
C1—B3—B7—B12	−61.82 (16)	B6—C1—C2—B7	−107.80 (14)
C1—B3—B8—B4	−39.12 (11)	B6—C1—C2—B11	−38.34 (13)
C1—B3—B8—B7	98.22 (13)	B6—C1—B3—C2	31.37 (12)
C1—B3—B8—B9	−1.92 (17)	B6—C1—B3—B4	−105.08 (14)
C1—B3—B8—B12	61.45 (15)	B6—C1—B3—B7	−3.25 (17)
C1—B4—B5—B6	34.71 (12)	B6—C1—B3—B8	−65.28 (16)
C1—B4—B5—B9	134.96 (13)	B6—C1—B4—B3	104.84 (14)
C1—B4—B5—B10	97.73 (13)	B6—C1—B4—B5	−37.07 (13)
C1—B4—B8—B3	39.91 (12)	B6—C1—B4—B8	65.00 (15)
C1—B4—B8—B7	1.53 (17)	B6—C1—B4—B9	2.23 (16)
C1—B4—B8—B9	−98.53 (13)	B6—C1—B5—B4	141.27 (13)
C1—B4—B8—B12	−61.69 (15)	B6—C1—B5—B9	102.08 (14)
C1—B4—B9—B5	−38.56 (11)	B6—C1—B5—B10	39.17 (12)
C1—B4—B9—B8	99.67 (13)	B6—C1—C11—C12	−59.64 (19)
C1—B4—B9—B10	−1.49 (16)	B6—C1—C11—C16	117.44 (16)
C1—B4—B9—B12	62.52 (15)	B6—C2—B3—C1	−32.51 (13)
C1—B5—B6—C2	36.54 (11)	B6—C2—B3—B4	4.73 (18)
C1—B5—B6—B10	135.11 (13)	B6—C2—B3—B7	106.83 (15)
C1—B5—B6—B11	96.93 (13)	B6—C2—B3—B8	66.60 (15)
C1—B5—B9—B4	38.86 (12)	B6—C2—B7—B3	−107.29 (14)
C1—B5—B9—B8	1.55 (17)	B6—C2—B7—B8	−66.86 (17)
C1—B5—B9—B10	−99.78 (13)	B6—C2—B7—B11	36.25 (13)
C1—B5—B9—B12	−62.32 (16)	B6—C2—B7—B12	−3.95 (18)
C1—B5—B10—B6	−39.27 (12)	B6—C2—B11—B7	−143.06 (13)
C1—B5—B10—B9	98.67 (13)	B6—C2—B11—B10	−40.46 (12)
C1—B5—B10—B11	−1.13 (17)	B6—C2—B11—B12	−103.15 (14)
C1—B5—B10—B12	61.81 (16)	B6—B5—B9—B4	101.33 (13)
C1—B6—B10—B5	38.42 (11)	B6—B5—B9—B8	64.01 (16)
C1—B6—B10—B9	0.97 (16)	B6—B5—B9—B10	−37.31 (12)
C1—B6—B10—B11	−98.94 (13)	B6—B5—B9—B12	0.15 (17)
C1—B6—B10—B12	−61.87 (16)	B6—B5—B10—B9	137.94 (13)
C1—B6—B11—C2	−34.17 (11)	B6—B5—B10—B11	38.14 (13)
C1—B6—B11—B7	−1.71 (17)	B6—B5—B10—B12	101.08 (14)
C1—B6—B11—B10	99.06 (13)	B6—B10—B11—C2	40.29 (12)
C1—B6—B11—B12	61.50 (15)	B6—B10—B11—B7	100.88 (14)
C1—C11—C12—F12	−3.8 (2)	B6—B10—B11—B12	138.35 (14)
C1—C11—C12—C13	175.70 (15)	B6—B10—B12—B7	0.34 (18)
C1—C11—C16—C15	−177.46 (15)	B6—B10—B12—B8	63.34 (16)
C1—C11—C16—F16	2.2 (2)	B6—B10—B12—B9	100.62 (14)
C2—C1—B3—B4	−136.45 (13)	B6—B10—B12—B11	−37.30 (12)
C2—C1—B3—B7	−34.62 (11)	B6—B11—B12—B7	−100.86 (14)
C2—C1—B3—B8	−96.65 (13)	B6—B11—B12—B8	−63.82 (16)
C2—C1—B4—B3	39.38 (12)	B6—B11—B12—B9	−0.51 (17)
C2—C1—B4—B5	−102.53 (13)	B6—B11—B12—B10	37.12 (12)
C2—C1—B4—B8	−0.46 (16)	B7—C2—B3—C1	−139.34 (13)
C2—C1—B4—B9	−63.23 (14)	B7—C2—B3—B4	−102.11 (13)
C2—C1—B5—B4	101.79 (14)	B7—C2—B3—B8	−40.23 (12)
C2—C1—B5—B6	−39.48 (12)	B7—C2—B6—C1	103.31 (15)
C2—C1—B5—B9	62.60 (15)	B7—C2—B6—B5	66.33 (16)
C2—C1—B5—B10	−0.31 (17)	B7—C2—B6—B10	4.15 (17)
C2—C1—B6—B5	136.52 (13)	B7—C2—B6—B11	−36.54 (14)
C2—C1—B6—B10	97.15 (13)	B7—C2—B11—B6	143.06 (13)
C2—C1—B6—B11	34.51 (12)	B7—C2—B11—B10	102.61 (14)
C2—C1—C11—C12	9.6 (2)	B7—C2—B11—B12	39.91 (12)
C2—C1—C11—C16	−173.29 (14)	B7—B3—B4—C1	−96.50 (13)
C2—B3—B4—C1	−36.56 (11)	B7—B3—B4—B5	−62.89 (16)
C2—B3—B4—B5	−2.95 (17)	B7—B3—B4—B8	37.87 (12)
C2—B3—B4—B8	97.81 (13)	B7—B3—B4—B9	0.51 (17)
C2—B3—B4—B9	60.45 (15)	B7—B3—B8—B4	−137.34 (13)
C2—B3—B7—B8	−133.39 (13)	B7—B3—B8—B9	−100.14 (14)
C2—B3—B7—B11	−32.40 (12)	B7—B3—B8—B12	−36.78 (12)
C2—B3—B7—B12	−96.22 (14)	B7—B8—B9—B4	−101.31 (14)
C2—B3—B8—B4	−98.20 (12)	B7—B8—B9—B5	−63.66 (16)
C2—B3—B8—B7	39.14 (11)	B7—B8—B9—B10	−0.21 (18)
C2—B3—B8—B9	−61.01 (15)	B7—B8—B9—B12	37.39 (13)
C2—B3—B8—B12	2.36 (15)	B7—B8—B12—B9	−137.90 (14)
C2—B6—B10—B5	97.77 (13)	B7—B8—B12—B10	−100.52 (14)
C2—B6—B10—B9	60.32 (15)	B7—B8—B12—B11	−37.33 (12)
C2—B6—B10—B11	−39.59 (12)	B7—B11—B12—B8	37.04 (12)
C2—B6—B10—B12	−2.52 (16)	B7—B11—B12—B9	100.35 (14)
C2—B6—B11—B7	32.45 (12)	B7—B11—B12—B10	137.98 (14)
C2—B6—B11—B10	133.22 (14)	B8—B3—B4—C1	−134.37 (13)
C2—B6—B11—B12	95.67 (14)	B8—B3—B4—B5	−100.76 (15)
C2—B7—B8—B3	−40.30 (12)	B8—B3—B4—B9	−37.35 (13)
C2—B7—B8—B4	−1.99 (17)	B8—B3—B7—C2	133.39 (13)
C2—B7—B8—B9	61.26 (16)	B8—B3—B7—B11	100.99 (14)
C2—B7—B8—B12	98.65 (14)	B8—B3—B7—B12	37.17 (13)
C2—B7—B11—B6	−32.94 (12)	B8—B4—B5—C1	−97.42 (13)
C2—B7—B11—B10	−96.00 (14)	B8—B4—B5—B6	−62.71 (16)
C2—B7—B11—B12	−133.85 (13)	B8—B4—B5—B9	37.53 (12)
C2—B7—B12—B8	−98.91 (14)	B8—B4—B5—B10	0.31 (17)
C2—B7—B12—B9	−61.44 (16)	B8—B4—B9—B5	−138.23 (13)
C2—B7—B12—B10	2.03 (17)	B8—B4—B9—B10	−101.17 (14)
C2—B7—B12—B11	39.45 (12)	B8—B4—B9—B12	−37.15 (13)
C2—B11—B12—B7	−39.07 (12)	B8—B7—B11—C2	96.17 (14)
C2—B11—B12—B8	−2.03 (16)	B8—B7—B11—B6	63.23 (16)
C2—B11—B12—B9	61.28 (15)	B8—B7—B11—B10	0.17 (18)
C2—B11—B12—B10	98.91 (13)	B8—B7—B11—B12	−37.68 (12)
B3—C1—C2—B6	146.76 (13)	B8—B7—B12—B9	37.47 (13)
B3—C1—C2—B7	38.96 (13)	B8—B7—B12—B10	100.94 (14)
B3—C1—C2—B11	108.42 (14)	B8—B7—B12—B11	138.36 (13)
B3—C1—B4—B5	−141.90 (13)	B8—B9—B10—B5	−100.80 (14)
B3—C1—B4—B8	−39.84 (12)	B8—B9—B10—B6	−63.10 (17)
B3—C1—B4—B9	−102.61 (13)	B8—B9—B10—B11	0.31 (18)
B3—C1—B5—B4	36.27 (13)	B8—B9—B10—B12	37.56 (13)
B3—C1—B5—B6	−105.00 (14)	B8—B9—B12—B7	−37.31 (13)
B3—C1—B5—B9	−2.92 (17)	B8—B9—B12—B10	−138.30 (14)
B3—C1—B5—B10	−65.83 (16)	B8—B9—B12—B11	−100.85 (14)
B3—C1—B6—C2	−31.35 (12)	B9—B4—B5—C1	−134.96 (13)
B3—C1—B6—B5	105.17 (14)	B9—B4—B5—B6	−100.25 (14)
B3—C1—B6—B10	65.80 (16)	B9—B4—B5—B10	−37.23 (12)
B3—C1—B6—B11	3.16 (17)	B9—B4—B8—B3	138.43 (13)
B3—C1—C11—C12	80.50 (19)	B9—B4—B8—B7	100.05 (14)
B3—C1—C11—C16	−102.42 (17)	B9—B4—B8—B12	36.83 (12)
B3—C2—B6—C1	32.57 (13)	B9—B5—B6—C1	−97.39 (13)
B3—C2—B6—B5	−4.42 (17)	B9—B5—B6—C2	−60.85 (15)
B3—C2—B6—B10	−66.59 (16)	B9—B5—B6—B10	37.72 (12)
B3—C2—B6—B11	−107.29 (15)	B9—B5—B6—B11	−0.46 (17)
B3—C2—B7—B8	40.43 (12)	B9—B5—B10—B6	−137.94 (13)
B3—C2—B7—B11	143.54 (13)	B9—B5—B10—B11	−99.80 (14)
B3—C2—B7—B12	103.34 (13)	B9—B5—B10—B12	−36.86 (12)
B3—C2—B11—B6	107.02 (14)	B9—B8—B12—B7	137.90 (14)
B3—C2—B11—B7	−36.04 (12)	B9—B8—B12—B10	37.38 (12)
B3—C2—B11—B10	66.56 (16)	B9—B8—B12—B11	100.58 (14)
B3—C2—B11—B12	3.86 (17)	B9—B10—B11—C2	−60.88 (16)
B3—B4—B5—C1	−34.20 (12)	B9—B10—B11—B6	−101.18 (14)
B3—B4—B5—B6	0.50 (17)	B9—B10—B11—B7	−0.30 (18)
B3—B4—B5—B9	100.75 (14)	B9—B10—B11—B12	37.17 (13)
B3—B4—B5—B10	63.53 (16)	B9—B10—B12—B7	−100.28 (14)
B3—B4—B8—B7	−38.38 (12)	B9—B10—B12—B8	−37.29 (12)
B3—B4—B8—B9	−138.43 (13)	B9—B10—B12—B11	−137.92 (13)
B3—B4—B8—B12	−101.60 (14)	B10—B5—B6—C1	−135.11 (13)
B3—B4—B9—B5	−101.17 (14)	B10—B5—B6—C2	−98.57 (13)
B3—B4—B9—B8	37.07 (12)	B10—B5—B6—B11	−38.18 (12)
B3—B4—B9—B10	−64.10 (16)	B10—B5—B9—B4	138.64 (13)
B3—B4—B9—B12	−0.08 (17)	B10—B5—B9—B8	101.33 (14)
B3—B7—B8—B4	38.31 (12)	B10—B5—B9—B12	37.46 (12)
B3—B7—B8—B9	101.56 (14)	B10—B6—B11—C2	−133.22 (14)
B3—B7—B8—B12	138.95 (13)	B10—B6—B11—B7	−100.77 (14)
B3—B7—B11—C2	32.75 (12)	B10—B6—B11—B12	−37.56 (13)
B3—B7—B11—B6	−0.19 (17)	B10—B9—B12—B7	100.99 (14)
B3—B7—B11—B10	−63.25 (17)	B10—B9—B12—B8	138.30 (14)
B3—B7—B11—B12	−101.10 (14)	B10—B9—B12—B11	37.45 (12)
B3—B7—B12—B8	−36.76 (13)	B10—B11—B12—B7	−137.98 (14)
B3—B7—B12—B9	0.71 (18)	B10—B11—B12—B8	−100.94 (14)
B3—B7—B12—B10	64.18 (17)	B10—B11—B12—B9	−37.62 (12)
B3—B7—B12—B11	101.60 (14)	C11—C1—C2—B3	109.06 (15)
B3—B8—B9—B4	−37.40 (12)	C11—C1—C2—B6	−104.18 (15)
B3—B8—B9—B5	0.26 (18)	C11—C1—C2—B7	148.02 (14)
B3—B8—B9—B10	63.71 (17)	C11—C1—C2—B11	−142.52 (14)
B3—B8—B9—B12	101.31 (15)	C11—C1—B3—C2	−109.50 (15)
B3—B8—B12—B7	37.09 (12)	C11—C1—B3—B4	114.05 (16)
B3—B8—B12—B9	−100.81 (14)	C11—C1—B3—B7	−144.12 (14)
B3—B8—B12—B10	−63.42 (16)	C11—C1—B3—B8	153.86 (14)
B3—B8—B12—B11	−0.23 (17)	C11—C1—B4—B3	−108.18 (15)
B4—C1—C2—B3	−39.81 (12)	C11—C1—B4—B5	109.92 (15)
B4—C1—C2—B6	106.95 (13)	C11—C1—B4—B8	−148.02 (14)
B4—C1—C2—B7	−0.85 (17)	C11—C1—B4—B9	149.21 (13)
B4—C1—C2—B11	68.61 (16)	C11—C1—B5—B4	−114.20 (16)
B4—C1—B3—C2	136.45 (13)	C11—C1—B5—B6	104.53 (15)
B4—C1—B3—B7	101.83 (14)	C11—C1—B5—B9	−153.40 (14)
B4—C1—B3—B8	39.80 (12)	C11—C1—B5—B10	143.70 (14)
B4—C1—B5—B6	−141.27 (13)	C11—C1—B6—C2	111.15 (14)
B4—C1—B5—B9	−39.19 (12)	C11—C1—B6—B5	−112.33 (14)
B4—C1—B5—B10	−102.10 (14)	C11—C1—B6—B10	−151.70 (13)
B4—C1—B6—C2	−99.18 (14)	C11—C1—B6—B11	145.66 (13)
B4—C1—B6—B5	37.34 (13)	C11—C12—C13—F13	−178.03 (14)
B4—C1—B6—B10	−2.04 (17)	C11—C12—C13—C14	2.1 (2)
B4—C1—B6—B11	−64.68 (16)	B11—C2—B3—C1	−103.11 (14)
B4—C1—C11—C12	153.91 (15)	B11—C2—B3—B4	−65.87 (16)
B4—C1—C11—C16	−29.0 (2)	B11—C2—B3—B7	36.23 (13)
B4—B3—B7—C2	95.46 (13)	B11—C2—B3—B8	−4.00 (16)
B4—B3—B7—B8	−37.93 (12)	B11—C2—B6—C1	139.86 (13)
B4—B3—B7—B11	63.06 (16)	B11—C2—B6—B5	102.87 (14)
B4—B3—B7—B12	−0.76 (17)	B11—C2—B6—B10	40.70 (12)
B4—B3—B8—B7	137.34 (13)	B11—C2—B7—B3	−143.54 (13)
B4—B3—B8—B9	37.20 (12)	B11—C2—B7—B8	−103.11 (14)
B4—B3—B8—B12	100.56 (14)	B11—C2—B7—B12	−40.20 (13)
B4—B5—B6—C1	−34.37 (12)	B11—B6—B10—B5	137.36 (13)
B4—B5—B6—C2	2.17 (16)	B11—B6—B10—B9	99.91 (14)
B4—B5—B6—B10	100.74 (14)	B11—B6—B10—B12	37.08 (13)
B4—B5—B6—B11	62.56 (16)	B11—B7—B8—B3	−101.53 (14)
B4—B5—B9—B8	−37.31 (13)	B11—B7—B8—B4	−63.22 (17)
B4—B5—B9—B10	−138.64 (13)	B11—B7—B8—B9	0.02 (17)
B4—B5—B9—B12	−101.18 (14)	B11—B7—B8—B12	37.41 (13)
B4—B5—B10—B6	−101.08 (13)	B11—B7—B12—B8	−138.36 (13)
B4—B5—B10—B9	36.86 (12)	B11—B7—B12—B9	−100.89 (14)
B4—B5—B10—B11	−62.94 (17)	B11—B7—B12—B10	−37.42 (13)
B4—B5—B10—B12	0.00 (17)	B11—B10—B12—B7	37.64 (13)
B4—B8—B9—B5	37.66 (13)	B11—B10—B12—B8	100.63 (14)
B4—B8—B9—B10	101.10 (14)	B11—B10—B12—B9	137.92 (13)
B4—B8—B9—B12	138.70 (14)	F12—C12—C13—F13	1.5 (2)
B4—B8—B12—B7	101.01 (14)	F12—C12—C13—C14	−178.38 (14)
B4—B8—B12—B9	−36.89 (12)	C12—C11—C16—C15	−0.1 (2)
B4—B8—B12—B10	0.50 (17)	C12—C11—C16—F16	179.54 (14)
B4—B8—B12—B11	63.69 (16)	C12—C13—C14—C15	−0.8 (2)
B4—B9—B10—B5	−37.37 (12)	C12—C13—C14—C141	178.50 (15)
B4—B9—B10—B6	0.33 (17)	B12—B7—B8—B3	−138.95 (13)
B4—B9—B10—B11	63.74 (17)	B12—B7—B8—B4	−100.64 (14)
B4—B9—B10—B12	100.99 (14)	B12—B7—B8—B9	−37.39 (13)
B4—B9—B12—B7	−0.39 (18)	B12—B7—B11—C2	133.85 (13)
B4—B9—B12—B8	36.92 (12)	B12—B7—B11—B6	100.91 (14)
B4—B9—B12—B10	−101.38 (14)	B12—B7—B11—B10	37.85 (13)
B4—B9—B12—B11	−63.93 (16)	B12—B8—B9—B4	−138.70 (14)
B5—C1—C2—B3	−106.66 (13)	B12—B8—B9—B5	−101.05 (14)
B5—C1—C2—B6	40.10 (12)	B12—B8—B9—B10	−37.60 (13)
B5—C1—C2—B7	−67.70 (16)	B12—B9—B10—B5	−138.37 (13)
B5—C1—C2—B11	1.76 (17)	B12—B9—B10—B6	−100.67 (14)
B5—C1—B3—C2	99.73 (14)	B12—B9—B10—B11	−37.25 (13)
B5—C1—B3—B4	−36.72 (13)	B12—B10—B11—C2	−98.05 (14)
B5—C1—B3—B7	65.11 (16)	B12—B10—B11—B6	−138.35 (14)
B5—C1—B3—B8	3.09 (17)	B12—B10—B11—B7	−37.46 (13)
B5—C1—B4—B3	141.90 (13)	F13—C13—C14—C15	179.35 (14)
B5—C1—B4—B8	102.07 (14)	F13—C13—C14—C141	−1.4 (2)
B5—C1—B4—B9	39.29 (12)	C13—C14—C15—F15	179.88 (15)
B5—C1—B6—C2	−136.52 (13)	C13—C14—C15—C16	−0.9 (2)
B5—C1—B6—B10	−39.37 (12)	C13—C14—C141—F17	61.8 (2)
B5—C1—B6—B11	−102.01 (13)	C13—C14—C141—F18	−56.8 (2)
B5—C1—C11—C12	−130.74 (16)	C13—C14—C141—F19	−176.85 (15)
B5—C1—C11—C16	46.3 (2)	C14—C15—C16—C11	1.4 (3)
B5—B4—B8—B3	101.10 (14)	C14—C15—C16—F16	−178.31 (15)
B5—B4—B8—B7	62.72 (16)	F15—C15—C16—C11	−179.38 (15)
B5—B4—B8—B9	−37.33 (12)	F15—C15—C16—F16	1.0 (2)
B5—B4—B8—B12	−0.50 (17)	C15—C14—C141—F17	−119.01 (19)
B5—B4—B9—B8	138.23 (13)	C15—C14—C141—F18	122.40 (19)
B5—B4—B9—B10	37.07 (12)	C15—C14—C141—F19	2.4 (2)
B5—B4—B9—B12	101.08 (14)	C16—C11—C12—F12	178.93 (14)
B5—B6—B10—B9	−37.45 (12)	C16—C11—C12—C13	−1.6 (2)
B5—B6—B10—B11	−137.36 (13)	C141—C14—C15—F15	0.7 (3)
B5—B6—B10—B12	−100.28 (14)	C141—C14—C15—C16	179.88 (16)

### Hydrogen-bond geometry (Å, º)

**Table:**

*D*—H···*A*	*D*—H	H···*A*	*D*···*A*	*D*—H···*A*
C2—H2···F12	0.91 (2)	2.11 (2)	2.7436 (19)	126 (2)
